# Computer-aided facial analysis as a tool to identify patients with Silver–Russell syndrome and Prader–Willi syndrome

**DOI:** 10.1007/s00431-023-04937-x

**Published:** 2023-03-22

**Authors:** Silvia Ciancia, Wesley J. Goedegebuure, Lionne N. Grootjen, Anita C. S. Hokken-Koelega, Gerthe F. Kerkhof, Daniëlle C. M. van der Kaay

**Affiliations:** 1 Department of Pediatrics, Subdivision of Endocrinology, Erasmus University Medical Center, Sophia Children’s Hospital, Rotterdam, Netherlands; 2 Post-Graduate School of Pediatrics, Department of Medical and Surgical Sciences for Mothers, Children and Adults, University of Modena and Reggio Emilia, Modena, Italy

**Keywords:** Silver–Russell syndrome, Prader–Willi syndrome, Face2Gene, Gestalt, Computer-aided facial analysis

## Abstract

Genetic syndromes often show facial features that provide clues for the diagnosis. However, memorizing these features is a challenging task for clinicians. In the last years, the app Face2Gene proved to be a helpful support for the diagnosis of genetic diseases by analyzing features detected in one or more facial images of affected individuals. Our aim was to evaluate the performance of the app in patients with Silver–Russell syndrome (SRS) and Prader–Willi syndrome (PWS). We enrolled 23 pediatric patients with clinically or genetically diagnosed SRS and 29 pediatric patients with genetically confirmed PWS. One frontal photo of each patient was acquired. Top 1, top 5, and top 10 sensitivities were analyzed. Correlation with the specific genetic diagnosis was investigated. When available, photos of the same patient at different ages were compared. In the SRS group, Face2Gene showed top 1, top 5, and top 10 sensitivities of 39%, 65%, and 91%, respectively. In 41% of patients with genetically confirmed SRS, SRS was the first syndrome suggested, while in clinically diagnosed patients, SRS was suggested as top 1 in 33% of cases (*p* = 0.74). Face2Gene performed better in younger patients with SRS: in all patients in whom a photo taken at a younger age than the age of enrollment was available, SRS was suggested as top 1, albeit with variable degree of probability. In the PWS group, the top 1, top 5, and top 10 sensitivities were 76%, 97%, and 100%, respectively. PWS was suggested as top 1 in 83% of patients genetically diagnosed with paternal deletion of chromosome 15q11-13 and in 60% of patients presenting with maternal uniparental disomy of chromosome 15 (*p* = 0.17). The performance was uniform throughout the investigated age range (1–15 years).

*Conclusion*: In addition to a thorough medical history and detailed clinical examination, the Face2Gene app can be a useful tool to support clinicians in identifying children with a potential diagnosis of SRS or PWS.

**Table:** 

**What is Known:** • *Several genetic syndromes present typical facial features that may provide clues for the diagnosis.* • *Memorizing all syndromic facial characteristics is a challenging task for clinicians.*	
**What is New:** • *Face2Gene may represent a useful support for pediatricians for the diagnosis of genetic syndromes.* • *Face2Gene app can be a useful tool to integrate in the diagnostic path of patients with SRS and PWS.*

**What is Known:**

• *Several genetic syndromes present typical facial features that may provide clues for the diagnosis.*

• *Memorizing all syndromic facial characteristics is a challenging task for clinicians.*

**What is New:**

• *Face2Gene may represent a useful support for pediatricians for the diagnosis of genetic syndromes.*

• *Face2Gene app can be a useful tool to integrate in the diagnostic path of patients with SRS and PWS.*

## Introduction

Genetic conditions affect between 5 and 8% of the population [1, 2], and approximately 30–40% show recognizable facial features that provide clues for the diagnosis [3]. Memorizing syndromic facial characteristics is a challenging task for clinicians because of the high number of different syndromes and the fact that various syndromes share similar facial features. In recent years, advances in artificial intelligence have allowed to develop tools like Face2Gene (FDNA, Inc., Boston, MA, USA) as an aid in recognizing the most common syndromes based on facial gestalt. Face2Gene is based on DeepGestalt technology, where gestalt refers to the information contained in the facial morphology. DeepGestalt technology, based on computer vision and deep learning algorithms, quantifies similarities of hundreds of known syndromes and provides a list of suggested syndromes [4].

Silver–Russell syndrome (SRS) and Prader–Willi syndrome (PWS) are two examples of disorders for which early recognition is of utmost importance for proper treatment and support.

SRS is an imprinting disorder for which an underlying genetic cause is found in approximately 60% of patients who are clinically diagnosed based on the Netchine–Harbison scoring system [5]. The two main genetic alterations are loss of methylation (LOM) of chromosome 11p15 (30–60% of patients) and maternal uniparental disomy of chromosome 7 (UPD(7)mat; 5–10% of patients). Patients with SRS can present with being born small for gestational age, postnatal growth failure, feeding difficulties in the first years of life, low body mass index (BMI), body asymmetry, and typical facial features: a broad forehead tapering into a small, pointed chin giving the triangular face characteristic; a slightly beaked nose with a prominent nasal bridge; a well-demarcated philtrum; a wide mouth with downturned edges; lips with a thin vermilion border, especially the upper lip; micrognathia; and posteriorly rotated and somewhat low-set ears. These facial features become less obvious with age, making the diagnosis more difficult in older patients [6, 7].

PWS is caused by the lack of expression of genes on the paternally inherited chromosome 15q11.2-q13 region. There are three main genetic subtypes in PWS: paternal 15q11-q13 deletion (50–55% of patients), maternal uniparental disomy of chromosome 15 (UPD(15)mat; 45–50% of patients), and imprinting defects of the aforementioned region (1–3% of patients) [8]. Newborns with PWS present with hypotonia, poor sucking, and subsequent failure to thrive. In early childhood, children with PWS have short stature, food seeking behavior with excessive weight gain and early obesity when food intake is not restricted, developmental delay, cognitive disability, and behavioral problems. Patients with PWS have typical facial features which include a thin upper lip, a downturned mouth, a narrow nasal bridge, almond-shaped palpebral fissures, narrow bitemporal diameter, and strabismus. Although such features can be present at birth, they may not be obvious and progress slowly over time [9–12].

The aim of our study was to assess the clinical utility of Face2Gene technology in identifying patients with clinically or genetically proven SRS or PWS.

## Materials and methods

### Subjects

Our study group consisted of 23 children with a clinical or genetically confirmed diagnosis of SRS and 29 children with genetically confirmed PWS. The study was approved by the Medical Ethics Committee of the Erasmus Medical Center, and written informed consent was obtained from parents or guardians and children aged 12 years or older. Consent to use the photos as examples was given by both parents of the patients and the patient if aged 12 years or older.

### Clinical parameters

The following clinical characteristics were retrieved from the patients’ electronic health records: sex, age at diagnosis, age at enrolment in the study, genetic diagnosis, birth weight, birth length, head circumference at birth, gestational age, height at diagnosis, weight at diagnosis, and target height. Height, weight, and weight for height were expressed as standard deviation scores (SDS) for calendar age and sex [13].

### Facial analysis

A frontal photo was taken from each patient through the Face2Gene app that was downloaded on the smartphones of the investigators. Access to the app is protected by a personal password after individual subscription to the app. Original photos are immediately encrypted and stored securely in a separate area of the Face2Gene database which is only available to the individual clinician or researcher who submitted the case.

The app uses a face detection technology built on a cascaded DCNN-based method (deep convolutional neural networks). Landmarks are used to define multiple facial regions from which specific features are extracted and compared to the FDNA’s database, containing phenotypic and genotypic information from more than 10,000 syndromes [4].

We investigated the app performance by assessing the top 1, top 5, and top 10 sensitivities. Top 1 means that the correct syndrome is suggested as the first option of the list, while top 5 and top 10 mean that the correct syndrome is suggested among the first five syndromes and first ten syndromes, respectively. Initially, no clinical details were added. When the correct diagnosis was not provided in the first ten diagnoses, we added clinical information from the available list of dysmorphic features and medical information listed in the app. If the software was unable to provide the correct diagnosis after the additional clinical information, we considered it as a negative result. Also, we evaluated the gestalt similarity using the “gestalt level” shown as a bar plot indicating levels of “high,” “medium,” and “low” similarity. Finally, we evaluated the performance of the tool in differentiating patients with SRS who were clinically diagnosed versus patients with SRS who were genetically diagnosed assessing the top 1, top 5, and top 10 sensitivity for each group, and we compared the performance of the tool between the different genetic subtypes of PWS.

### Statistical analysis

Demographic characteristics were expressed as counts and percentages for categorical variables and mean (± SDS) for continuous variables. Categorical variables were compared using chi-square test. The top 1, top 5, and top 10 sensitivities were based on the percentage of patients for whom the correct syndrome (SRS or PWS) was suggested, respectively, as first option or among the first 5 or the first 10 options. The statistical analysis was performed with GraphPad Prism version 9.3.0. *p* values < 0.05 were considered statistically significant.

## Results

**Fig. 1:**
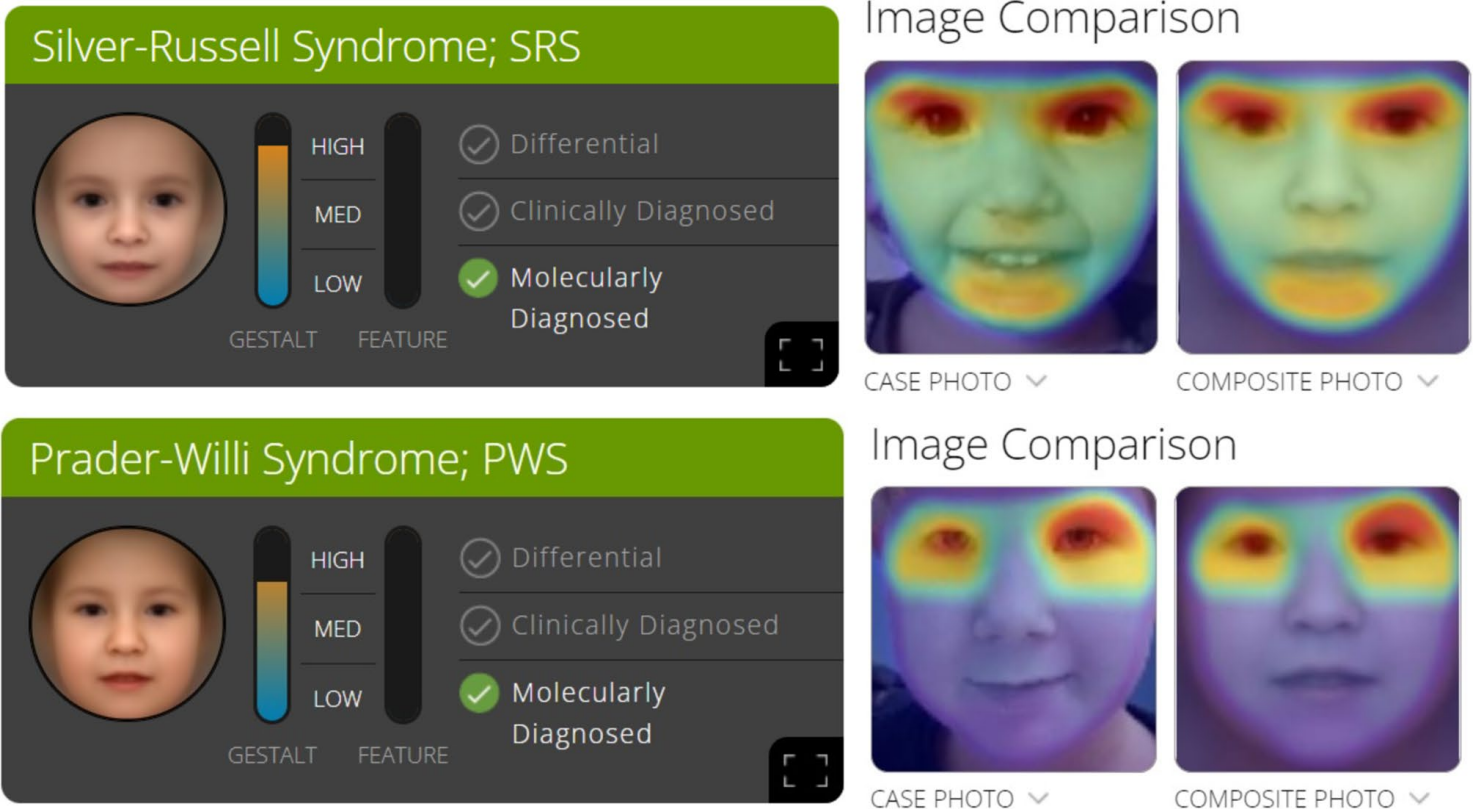
Face2Gene app feedback on an SRS and a PWS patient. On the left: SRS (above) and PWS (below) suggested with high probability in one patient for each cohort. Both patients were molecularly diagnosed. The composite photo is shown. It results from the photos of affected individuals uploaded in the database, and it is compared by the app with the case picture to score the probability that the patient is affected by SRS (above) and PWS (below) on the basis of facial features. On the right: the heat map marks the areas of resemblance between the photo of the patient (SRS picture above, PWS picture below) and the composite photo. The intensity of the color scale grades from light blue to red; the red areas are more suggestive for the analyzed syndrome

An example of the Face2Gene app feedback for a patient with SRS and a patient with PWS is shown in Fig. 1.

### Silver–Russell syndrome

Twenty-three patients were enrolled, 15 boys (65%) and 8 girls (35%). The mean age at enrollment in the study was 7 years (range 1–17 years). The diagnosis was genetically confirmed in 74% of patients. In 53% of these patients, the causative genetic mutation was 11p15 LOM, in 23.5% UPD(7)mat. Other genetic diagnoses were duplication of chromosome 11p15 (*N* = 2), mutation in the *HMGA2* gene (*N* = 1), and UPD(20)mat (*N* = 1). The mean age at diagnosis was 2.6 years; in 2 patients, the diagnosis was made prenatally (in one case with amniocentesis and in the other case due to intrauterine growth restriction in combination with a family history positive for SRS). Except for 1 patient who started growth hormone treatment at 1 month after enrollment in this study, all patients were treated with growth hormone. The median (interquartile range) duration of growth hormone treatment was 4.1 (7.0) years. Baseline characteristics at birth and at diagnosis are shown in Table 1.

**Table 1:** Baseline characteristics of SRS and PWS patients at birth and at diagnosis

		**SRS (23)**	**PWS (29)**
**At birth**	**Gestational age (weeks)**	35.3 (± 4.1)	38.7 (± 2.7)
	**Length *Z*-score**	− 2.6 (± 1.3)	− 1.4 (± 3.4)
	**Weight *Z*-score**	− 2.6 (± 1.1)	− 1.4 (± 0.8)
	**Head circumference *Z*-score**	− 1.2 (± 1.6)	− 0.1 (± 0.8)
	**Target height *Z*-score**	− 0.7 (± 0.6)	+ 0.4 (± 1.0)
**At diagnosis**	**Height *Z*-score**	− 3.6 (± 1.0)	N.A
	**Weight for height *Z*-score**	− 3.1 (± 1.8)	N.A

Data expressed as mean (± SDS). *SDS* Standard Deviation Score, *NA* Not Available

Face2Gene showed a top 1, top 5, and top 10 sensitivity of 39% (9 patients), 65% (15 patients), and 91% (21 patients), respectively. In 9% (2 patients), the diagnosis of SRS was not suggested among the first 10 options. This did not improve after adding clinical features (among the possible features listed in the app, only short stature was applicable to both patients). For these 2 patients with clinical SRS, the diagnosis was not genetically confirmed after extensive genetic investigations (methylation and CNV analysis chromosome of 6, 7, 11, 14, 15, 19, and 20 and next-generation sequencing of 18 short stature-related genes including *HMGA2*). Both patients fulfilled the clinical diagnosis of SRS based on 4 out of 6 criteria from the Netchine–Harbison clinical scoring system ((1) small for gestational age, (2) postnatal growth retardation, (3) severe feeding difficulties in early life with a BMI ≤ − 2 SDS, and (4) a protruding forehead at toddler age). Relative macrocephaly at birth and body asymmetry were absent in both. In Table 2, the fulfilling of the Netchine–Harbison criteria and the genetic analysis performed in all patients with a clinical diagnosis of SRS are reported.

**Table 2:** Netchine–Harbison (NHS) criteria and genetic analysis performed in 6 patients with clinical diagnosis of SRS

	Patient 1	Patient 2	Patient 3	Patient 4	Patient 5	Patient 6
NHS criteria (yes/no):						
SGA	Yes	Yes	Yes	Yes	Yes	Yes
Postnatal growth retardation	Yes	Yes	Yes	Yes	Yes	Yes
Relative macrocephaly at birth	No	Yes	No	No	No	Yes
Protruding forehead	Yes	Yes	Yes	Yes	Yes	Yes
Feeding difficulties and/or BMI ≤ −2 SDS	Yes	Yes	Yes	Yes	Yes	Yes
Body asymmetry	No	No	No	No	No	No
Genetic analysis (performed yes/no):						
Molecular testing 11p15 and UPD(7)mat	Yes	Yes	Yes	Yes	Yes	Yes
Chromosome microarray analysis	Yes	Yes	Yes	Yes	Yes	Yes
Multilocus methylation and CNV analysis^a^	Yes	Yes	No	Yes	No	Yes
NGS short stature-related genes^b^	Yes	Yes	Yes	Yes	Yes	No
Face2Gene result:						
Age at enrollment (years)	3	14.4	9.6	6.8	13.3	2.8
Position SRS diagnosis^c^	7	2	1	No top 10	No top 10	1
Gestalt level	Low	Low	Low	-	-	Medium

*SGA* small for gestational age

^a^Methylation and CNV analysis of chromosome 6, 7, 11, 14, 15, 19, and 20

^b^Next-generation sequencing of 18 short stature-related genes including *HMGA2*

^c^Face2Gene ranks a list of syndromes from the most to the least likely for each picture analyzed. The numbers in the table indicate the position of SRS in this list. For each syndrome, the gestalt similarity with suggested syndromes is estimated and divided into three different levels of probability (low, medium, and high)

Of the 91% of patients with SRS for whom Face2Gene suggested the syndrome among the first 10 options, 13% had SRS suggested with high level of probability, 30% with medium level of probability, and 48% with low level of probability. Among patients in whom SRS was suggested with low probability, one patient (9%) had the syndrome as top 1 suggestion, while in 91% of patients, SRS was suggested in the top 5 or top 10. The 3 patients with SRS suggested as top 1 and with high probability were young (2, 2.8, and 3 years old), and all had genetic confirmation of SRS (11p15 LOM and UPD(7)mat).

A photo at diagnosis was obtained from 3 patients, while for 2 others, it was possible to obtain a photo at a younger age than the age at enrollment in this study. Face2Gene had a better performance for photos obtained at a younger age: for all 5 patients, SRS was suggested as top 1, with variable degree of probability. In one patient, SRS was suggested with higher probability for the photo taken at the age of enrollment (age 2 years and 10 months), likely because the quality of the photo obtained at a younger age (age 14 months) was lower (Table 3).

**Table 3:** Influence of age on Face2Gene results in 5 SRS patients in whom a picture at a younger age than at enrollment was available

	**Age at enrollment**	**Face2Gene analysis**	**Picture at younger age**	**Face2Gene analysis**
Patient 1	12 years	7, very low	4 years	1, medium
Patient 2	2 years, 10 months	1, high	14 months	1, medium
Patient 3	2 years, 9 months	7, low	15 months	1, high
Patient 4	13 years	8, very low	1 year	1, low
Patient 5	16 years	9, low	5 years	1, medium

Each row refers to 1 patient; the results of the Face2Gene analysis are shown as ranking of SRS in the list provided by the app (from 1 to 10) and level of probability (from very low to high)

In 41% of patients with genetically confirmed SRS, SRS was the first syndrome suggested, while in clinically diagnosed patients, SRS was suggested as top 1 in 33% of cases (*p* = 0.74).

We found no statistically significant difference between the top 1 and top 5 sensitivities and the specific genetic diagnosis, in particular 11p15 LOM and UPD(7)mat (*p* = 0.85 and *p* = 0.52, respectively).

Lastly, we analyzed the list of the first five syndromes suggested for each patient as differential diagnosis: neurofibromatosis type 1 was suggested in 52% of patients, followed by Noonan syndrome (39%) and fetal alcohol syndrome (35%).

### Prader–Willi syndrome

Twenty-nine patients were enrolled, 15 boys (52%) and 14 girls (48%). The mean age at enrollment in the study was 7 years (range 1–15 years). Eighteen patients were genetically diagnosed with paternal deletion of chromosome 15 (62%), 10 patients with UPD(15)mat (34.5%), and 1 patient (3.5%) had a punctiform mutation of the imprinting center on chromosome 15. In 79% of patients, the diagnosis was made shortly after birth due to severe hypotonia; in 4 cases, the diagnosis was made during the first year of life; the oldest patient was 4.7 years at diagnosis. All patients except for 1 (due to preference of parents) were treated with growth hormone. The median (interquartile range) duration of GH treatment was 5.2 (8.1) years. Baseline characteristics at birth and at diagnosis are shown in Table 1.

Face2Gene showed a top 1, top 5, and top 10 sensitivity of 76%, 97%, and 100%, respectively. Twenty-one percent of patients with PWS had the syndrome suggested with high level of probability, 38% with medium level of probability, and 41% with low level of probability.

Among patients in whom PWS was suggested with low probability, 46% had the syndrome as top 1. In 92% of patients, PWS was suggested in the top 5 while for the remaining 8% in the top 10 diagnosis.

PWS was suggested as top 1 in 83% of patients who were genetically diagnosed with paternal deletion of chromosome 15 and in 60% of patients with UPD(15)mat (*p* = 0.17). Eighty percent of patients in whom PWS was suggested with high probability had paternal deletion of chromosome 15. PWS as first suggestion was equally distributed throughout the investigated age range.

Among the first five syndromes suggested as differential diagnosis for each patient, the 22q11.2 deletion syndrome was the most common (59% of patients), followed by Klinefelter syndrome (48% of patients, of whom 57% were female) and Angelman syndrome (45%).

## Discussion

In this study, we evaluated the usability of the Face2Gene app in identifying patients with SRS and PWS. The correct diagnosis was suggested among the top 10 in 91% of SRS patients and in 100% of PWS. Therefore, the sensitivity was in line with what was reported by Gurovich et al. [4] and comparable to studies performed in children with various syndromes such as Cornelia De Lange syndrome [14] and Angelman syndrome [15].

Face2Gene has been tested in patients affected by very rare syndromes [16, 17], with variable overall sensitivity. In the study of Mishima et al., the top 1 sensitivity ranged from 42.9 to 61.2% and the top 10 sensitivity from 60 to 85.7%. Patients affected by 48 different dysmorphic syndromes were tested; the higher sensitivities were found after excluding patients affected by syndromes not included in the Face2Gene database and for which the app was not trained. Similar results were found by Marwaha et al. (top 10 sensitivity 57%, increasing to 82% when patients with syndromes not included by Face2Gene were excluded). The app has been validated in non-Caucasian patients, showing a good ability in suggesting the diagnosis despite facial variations that occur in different ethnic groups [18–20].

For the 2 SRS patients in whom the app did not suggest SRS as a top 10 diagnosis, SRS was clinically diagnosed according to the Netchine–Harbison clinical scoring system but not confirmed after extensive genetic testing (multilocus methylation and CNV analysis and next-generation sequencing of 18 short stature-related genes including *HMGA2*). This SRS scoring system has a low specificity and thus could result in false-positive results, especially since in both patients, the criterion “relative macrocephaly at birth” was absent. On the other hand, the spectrum of SRS and SRS-like diagnoses is extending, and rare genetic causes in the 11p15.5 region such as *CDKN1C* mutations, loss-of-function of *IGF2*, and large copy number variants in this region have been described [21]. Disruptions in the *HMGA2-PLAG1-IGF2* pathway can cause an SRS phenotype [22]. In patients with a mutation in the *HMGA2* gene, microcephaly at birth has been described [23]. It might be possible that, in the coming years, the Face2Gene app can be trained to recognize facial features of patients with non-classical SRS, if it is used more extensively.

In the SRS cohort, the app performed better when a photo taken at a younger age was available. SRS was suggested as top 1 and with higher probability in almost all patients aged 5 years or younger. This can be explained by the fact that facial features suggestive of SRS are more evident in the first years of life [7].

Few studies investigated the influence of GH treatment on craniofacial dimensions in children [24]. It is suggested that facial convexity (balance between forehead and upper and lower jaw) decreases, and mandibular length and posterior facial height increase during GH treatment in patients with GH deficiency, idiopathic short stature, being born small for gestational age with persistent short stature, and various genetic disorders. Although this may influence the performance of Face2Gene, it was not feasible to determine if it might have been of influence in our study since this was a cross-sectional study.

In the PWS group, the app performance was good. PWS is usually diagnosed in the first weeks after birth because of severe hypotonia and feeding problems. However, in some cases, the neonatal phenotype can be milder or unrecognized, the choice of the molecular test inappropriate [25], or genetic tests not available. In these cases, the diagnosis could be delayed, and tools such as Face2Gene might be supportive. Although the typical facial features in PWS become more evident in older patients, in our cohort, the performance was homogeneously satisfactory throughout the tested age range (1–15 years). Further studies performed in infants could be useful to investigate the usability of Face2Gene in children with PWS in the first months of life.

Based on our study and other studies mentioned above [14–17], Face2Gene can be helpful to identify potential syndromes which can be challenging for clinicians. However, there are some potential caveats to consider. First, the app always shows a list of 30 syndromes, also in healthy subjects without any suspicion of a syndrome or disease. Secondary, the app can suggest syndromes that do not match the phenotype except for partial overlap of some facial features. Thus, the level of probability shown by the bar plot (low, medium, or high) is the most relevant result to consider, with a high level of gestalt similarity being more relevant than the top 1 suggestion with low probability. In the study of Pantel et al., the performance of Face2Gene was studied in 323 patients affected by genetic syndromes and 323 healthy controls matched for age, sex, and ethnicity. The authors reported significantly lower scores (levels of probability) for detecting a genetic syndrome in the control cohort [26].

If a syndrome is suggested with medium–high probability, the clinician must critically evaluate if the suggestion matches the phenotype of the patient. Consultation with medical specialists such as clinical geneticists is of utmost importance and should be considered early in the course of the diagnostic work-up.

Lastly, some syndromes are often suggested in the list, creating a so-called background noise. For example, Klinefelter syndrome was proposed among the top 5 suggestion in 48% of PWS patients, of whom 57% were female.

Face2Gene presents several favorable aspects that make its use convenient. It is free of charge, it can be easily downloaded on the smartphone of verified healthcare professionals, and the access is protected by a password. Photos can be taken quickly through the app, with the possibility of using the face capture (the camera automatically takes the photo when the app frames the face contour) or can be uploaded from the gallery. Original photos are encrypted and stored in a database available only to the clinician or researcher who submitted the case. Nevertheless, ethical and privacy concerns related to the use of facial recognition technology and big data have been raised [27]. A thorough and careful conversation with patients and/or their caregivers to obtain consent is, therefore, essential prior to the use of this tool.

Our study has some limitations. The relatively small number of patients in the subgroups (for example, patients with specific genetic findings) might have resulted in not being able to find statistically significant results. Due to the rarity of these syndromes, international multi-center studies would be necessary to obtain larger samples. A quality check of the photos, in order to confirm that the photo was taken from the right angle to properly analyze the face or if it was taken with appropriate light and contrast, was absent. We did not obtain photos at different ages for each patient in order to investigate more extensively the performance of the app at different ages. As the app was tested in patients that already received the diagnosis of SRS or PWS, it was not possible to calculate the positive predictive value of the tool. However, our data support the use of Face2Gene as a tool for the identification of patients affected by SRS or PWS. A delay in diagnosis could compromise the care for these patients and unnecessarily delay treatment with, for example, growth hormone.

In conclusion, our study demonstrates that the Face2Gene app can be a useful tool to integrate in the diagnostic path of patients with SRS and PWS, as support for the clinicians. Deep learning algorithms such as Face2Gene cannot substitute a clinician or a geneticist. Nevertheless, including its use in the diagnostic path of patients suspected to have a genetic syndrome can shorten the time needed to make the right diagnosis, may reduce healthcare costs by providing clues for more targeted genetic tests, and can be supportive in establishing clinical diagnoses in settings where genetic tests are not easily available [28]. Moreover, the algorithms allow improvement in the tool’s accuracy in the future, and the identification of (new) syndromes in various ethnic groups can be quickly shared among colleagues at an international level, to enhance a global and more equal access to knowledge.

## Data Availability

The data generated during this study are available from the corresponding author on reasonable request.

## Abbreviations

BMI: Body mass index
DCNN: Deep convolutional neural networks
GH: Growth hormone
LOM: Loss of methylation
PWS: Prader–Willi syndrome
SDS: Standard deviation scores
SRS: Silver–Russell syndrome
UPD(…)mat: Maternal uniparental disomy of chromosome
